# MRI-Derived Radiomics to Guide Post-operative Management for High-Risk Prostate Cancer

**DOI:** 10.3389/fonc.2019.00807

**Published:** 2019-08-27

**Authors:** Vincent Bourbonne, Martin Vallières, François Lucia, Laurent Doucet, Dimitris Visvikis, Valentin Tissot, Olivier Pradier, Mathieu Hatt, Ulrike Schick

**Affiliations:** ^1^Department of Radiation Oncology, University Hospital, Brest, France; ^2^LaTIM, INSERM, UMR 1101, Brest University, Brest, France; ^3^Université de Bretagne Occidentale, Brest, France; ^4^Medical Physics Unit, McGill University, Montreal, QC, Canada; ^5^Department of Anatomopathology, University Hospital, Brest, France; ^6^Department of Radiology, University Hospital, Brest, France

**Keywords:** magnetic resonance imaging, prostatic neoplasms, radiomics, machine learning, treatment failure

## Abstract

**Purpose:** Prostatectomy is one of the main therapeutic options for prostate cancer (PCa). Studies proved the benefit of adjuvant radiotherapy (aRT) on clinical outcomes, with more toxicities when compared to salvage radiotherapy. A better assessment of the likelihood of biochemical recurrence (BCR) would rationalize performing aRT. Our goal was to assess the prognostic value of MRI-derived radiomics on BCR for PCa with high recurrence risk.

**Methods:** We retrospectively selected patients with a high recurrence risk (T3a/b or T4 and/or R1 and/or Gleason score>7) and excluded patients with a post-operative PSA > 0.04 ng/mL or a lymph-node involvement. We extracted IBSI-compliant radiomic features (shape and first order intensity metrics, as well as second and third order textural features) from tumors delineated in T2 and ADC sequences. After random division (training and testing sets) and machine learning based feature reduction, a univariate and multivariate Cox regression analysis was performed to identify independent factors. The correlation with BCR was assessed using AUC and prediction of biochemical relapse free survival (bRFS) with a Kaplan-Meier analysis.

**Results:** One hundred seven patients were included. With a median follow-up of 52.0 months, 17 experienced BCR. In the training set, no clinical feature was correlated with BCR. One feature from ADC (SZE_GLSZM_) outperformed with an AUC of 0.79 and a HR 17.9 (*p* = 0.0001). Lower values of SZE_GLSZM_ are associated with more heterogeneous tumors. In the testing set, this feature remained predictive of BCR and bRFS (AUC 0.76, *p* = 0.0236).

**Conclusion:** One radiomic feature was predictive of BCR and bRFS after prostatectomy helping to guide post-operative management.

## Keypoints

– Texture analysis, based on prostatic MRI, provides an informative assessment of tumoral heterogeneity which could help to predict biochemical failure risk.
– Management of patients could be performed with a greater confidence.

## Introduction

Prostate cancer (PCa) is the most common cancer among men with ~165.000 patients diagnosed with the disease in 2017 in the United States, and more than 29.400 annual deaths (1). Radical prostatectomy (RP) is one of the treatments of choice for patients with PCa and is associated with excellent long-term outcomes. Nevertheless, biochemical recurrence (BCR) after RP occurs in 50% of patients, particularly in those who harbor high risk features like locally advanced disease (T3-4), positive margins (R1) or high Gleason score, and is predictive of metastatic relapse and cancer specific death (2). Adjuvant radiotherapy (aRT) of the prostatic bed has been proposed and proven to be effective in 3 randomized controlled trials (EORTC 22911, SWOG 8794, ARO 96-02) comparing aRT versus observation (3–6). All three studies showed a significant benefit for aRT in biochemical relapse-free survival (bRFS), but results were conflicting in terms of metastases-free and overall survival (6). In addition, patients receiving aRT experienced higher rates of grade 2 or higher gastrointestinal and genitourinary toxicities (5). Moreover, based on clinical and histopathological features alone, patient selection remains insufficient. In a multi-institutional study and after a 5-years follow-up (7), ~50% of the high–risk, operated on patients were still BCR-free and were without the certainty of the benefits from aRT. Therefore, radiation therapy (RT) is often delivered only at the time of BCR as it would then be limited solely to relapsing patients, and would reduce treatment-related side effects. Indeed, some data suggest that early salvage RT (sRT) is as efficient as aRT in this context (8). However, a low pretreatment serum prostate-specific antigen (PSA) level is known to be the strongest predictor of response after sRT, and the question remains as to whether sRT at the first time of recurrence compromises cancer control compared to aRT (9).

The natural history of relapse after radical prostatectomy (RP) is heterogeneous even in patients with high risk features and may reflect a broad range of underlying tumor pathophysiological processes. Recently, in addition to conventional parameters on magnetic resonance imaging (MRI) used to diagnose and stage cancer, there has been a growing interest in the high-throughput extraction of quantitative features from medical images, denoted radiomics. Radiomic features are statistical, geometrical, or textural metrics designed to quantify tumor intensity, shape and heterogeneity, which have been shown to reflect intratumorally histopathological properties and to provide prognostic information in several pathologies including PCa (10–12). For example, the GLSZM is a matrix focusing on the size of areas (or zones) of similar gray-level values. The more heterogeneous the intensities of the voxels in the tumor image are, the smaller the areas (or zones) of similar gray-level become, resulting in lower values of the GLSZM-based features.

An MRI-derived radiomics signature predictive of the outcome of patients after RP has not yet been described. We aimed to develop and validate such a signature with prognostic value in patients with high risk PCa, in order to guide the patients' selection and therapeutic management, especially regarding the use of aRT.

## Methods

### Patients Selection

All patients with histologically proven PCa patients treated with RP, with or without a lymphadenectomy from 2010 to 2016 at Brest, were retrospectively considered. Among them, those with high-risk features on the pathologic specimen, namely pT3a-b or pT4, and/or R1, and/or Gleason 8-10, and available preoperative pelvic MRI were retrospectively included.

All patients with lymph node involvement after extensive lymphadenectomy were excluded, as were those whose PCa diagnosis was obtained after cystoprostatectomy for bladder carcinoma. Patients who received adjuvant treatment (aRT and/or adjuvant androgen deprivation therapy) or those with post-operative PSA (PSA > 0.04 ng/mL at 3 months following RP) were also excluded.

All patients for which the MRI were not retrievable were excluded.

A follow-up of 24 months was mandatory, except in case of BCR.

### Outcome

The primary endpoint was the prediction of BCR, which was defined as a PSA increase above 0.2 ng/mL confirmed on two successive blood samples. The secondary endpoint was the prediction of bRFS.

### MRI

The MRI were performed on two different MRI scanners: a Phillips 3T (Philips Healthcare, The Netherlands) and a Siemens 1.5T (Siemens Healthcare, Malvern PA). Both scans were performed using a 6-channel phased-array surface coil. Patients were scanned in supine position. MRI sequences included axial turbo spin echo T2-weighted and axial diffusion sequences using multiple *b*-values (maximal *b*-value: 1,000 s/mm^2^), along with a perfusion sequence for Philips 3T and a T1 sequence with gadolinium injection for Siemens 1.5T. ADC maps were calculated using each corresponding manufacturer's software. MRI scans were performed according to ESUR guidelines. Full details about acquisition parameters are provided in the Table 1.

**Table 1:** Summary of MRI scan acquisition parameters.

**Acquisition parameters**	**Siemens 1.5T (*n* = 75)**	**Philips Achieva 3T (*n* = 32)**
Magnetic field strength (Tesla)	1.5T	3T
T2-Weighted		
Matrix (pixels)	192 × 192	268 × 268
Field of view (mm)	250 × 250	320 × 320
ET (ms)	110	90
RT (ms)	2,500	4,500
Slice Thickness (mm)	1.5	1.5
ADC map		
Matrix (pixels)	128 × 128	144 × 144
Field of view (mm)	200 × 200	240 × 240
ET (ms)	80	80
RT (ms)	2,300	2,300
Slice Thickness (mm)	3.5	3.5
Diffusion gradient	B50-400-1000	B100-600-1000

*RT, repetition time; ET, echo time*.

### Clinical Features

The following clinical variables were collected from medical records: size of the delineated tumor, T stage (extra-capsular extension, seminal vesicle invasion), Gleason score, pre- and post-operative PSA, margins status, age at surgery and the CAPRA-S Score (13). All categorical clinical features were remapped to ordinal values.

### Tumor Delineation

Prostatic tumors were semi-automatically delineated on all slices using the Fast GrowCut Effect extension available in 3D Slicer® v4.8.0, on both the ADC and T2-sequences using all sequences available on the pre-operative MRI (ADC, T2-weighted, diffusion, perfusion, T1 with gadolinium injection). An example is illustrated in Supplementary Figure 1.

### Radiomic Features

Prior to extraction of features, wavelet filters were applied to each MRI sequence. The high-pass and low-pass versions of the wavelet (14) basis function coiflet 1 were consecutively applied in the three directions of space, thereby creating eight filtered images: LLL, LLH, LHL, LHH, HLL, HLH, HHL, and HHH. Including the original image, nine images per MRI sequence were thus available for radiomics analysis. One hundred seventy-two radiomic features were extracted, using MathLab®, following the implementation guidelines defined by the Image Biomarker Standardization Initiative (IBSI) (15) workflow (Supplementary Figure 2). The textural radiomic features were implemented with different parametrization settings (see Supplementary Figure 2). As a result, the total available radiomic variables per MRI sequence per patient was 27,376.

### Statistical Analysis

The cohort was first randomly split into two sets, 2/3 for training (*n* = 70) and 1/3 for testing (*n* = 37). A machine learning workflow was subsequently employed to reduce this very large initial number of radiomic features to a relevant subset more suitable for robust statistical analysis. This selection was performed in the training set using an aggressive false discovery reduction procedure relying on stability checks, robustness score, and Pearson's correlation (PC) checks (16). More details about this procedure is provided below: The training set was sub-divided 100 times into different subsets with a 2:1 size ratio using stratified random sub-sampling. The PC of each radiomic feature with BCR was calculated for each of the 100 subsets. A given feature was considered stable if 95% of the absolute PC value were above 0.3. Following stability checks, the optimal extracted parameter was identified for each remaining feature in the set by maximizing the mean absolute PC, such that only one variant per feature was retained. Finally, intra-correlation between features still present in the set was analyzed and features with a coefficient >0.7 were discarded by prioritizing those with the highest PC.

Imbalanced distribution of the clinical outcome (BCR) was adjusted using the SMOTE technique (17) which was applied to the whole teaching set prior to the start of feature set reduction.

The reduced subset of radiomic features identified through the process described above, as well as all clinical variables, were then assessed for their predictive ability with univariate (ROC curves) and multivariate (Cox regression) analyses. Optimal cut-off values for each feature were defined via the Youden Index in the ROC curves. Based on additive combinations between each radiomic and clinical variable, three models were built and evaluated: radiomics-only, clinical-only, and radiomics combined with clinical. The performance of these models was evaluated using Kaplan-Meier curves and the log-rank test in the testing set.

To minimize the effects of variability between different types of scanners (1.5T vs. 3T), radiomics features were separately normalized (using z-score standardization, i.e., mean 0 and standard deviation 1) per scanner type and per training and testing set (16).

Finally, the predictive power of each model was then assessed on the overall population depending on the type of scan (1.5T vs. 3T).

Statistical analysis was performed using MedCalc v13.1.0.

### Ethical Considerations

This study was approved by the hospital ethical committee (PREBOP 29DRC18.0108) and all patients gave their consent for the use of their clinical and imaging data.

## Results

### Patients Characteristics

Between January 2010 and December 2016, 505 patients underwent RP ± extensive pelvic lymphadenectomy. According to pathological analysis, 272 patients (54%) presented high-risk features (T3a/T3b or T4, and/or R1, and/or Gleason 8-10).

**Figure 1:**
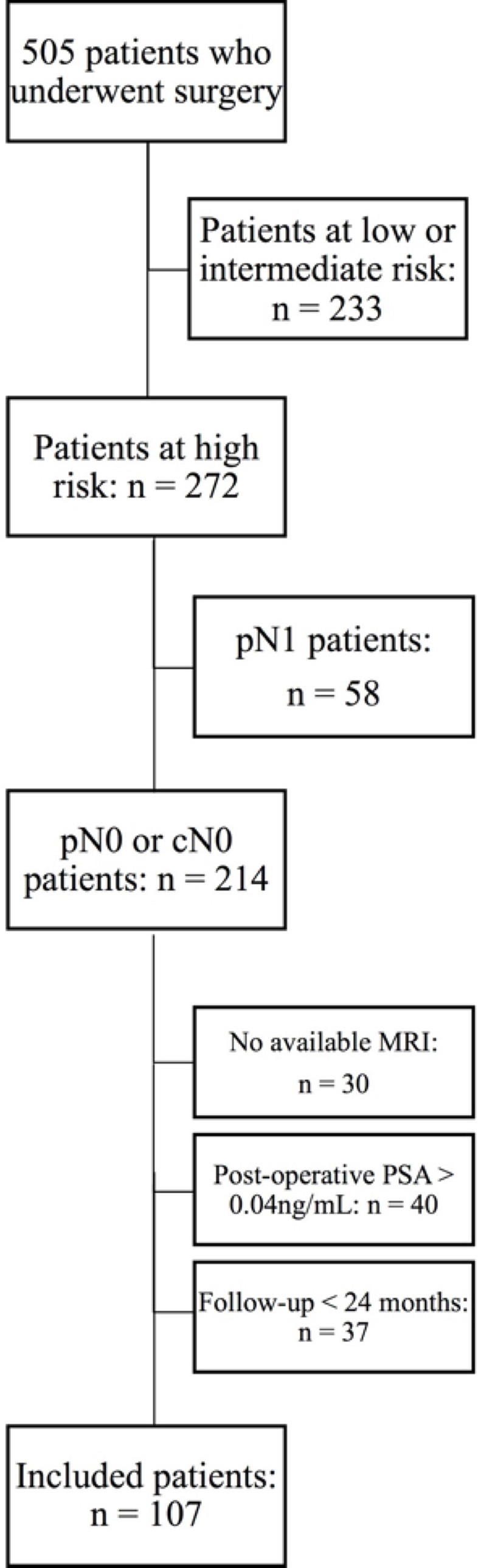
Flowchart of the patients selection.

Overall, 107 patients were excluded because of positive lymph nodes (*n* = 58), follow up <24 months (*n* = 37) or post-operative PSA >0.04 ng/mL (*n* = 40). Among the remaining patients (*n* = 137), preoperative MRI was available for 107 (78%). The flowchart of patients' selection is available as Figure 1.

Clinical and histopathological characteristics did not significantly differ between the training and testing sets (Table 2). A majority of patients had pT3 disease (65%) and microscopic involved margins (67%). No pT4 (0%) patients were finally included. Seventy percent of scans (*n* = 75) were acquired on the Siemens scanner and 30% (*n* = 32) on the Philips scanner (Table 1).

**Table 2:** Patients and tumors characteristics in training and testing sets.

**Patients characteristics**	**Training *N* = 70**	**Testing *N* = 37**	***p*-value**
Age at diagnosis (mean, y)	65	65	0.81
PSA (mean, ng/mL)	9	9	0.81
MRI characteristics			
Siemens 1.5T (%)	67	73	0.69
Philips 3T (%)	33	27	
Surgical characteristics			
Pathological tumor stage			
pT1-pT2 (%)	33	41	0.57
pT3 (%)	67	60	
pT4 (%)	0	0	
Nodal status			
pN0 (%)	85	78	0.56
cN0 (%)	15	22	
Surgical margins			
R0 (%)	41	41	0.91
R1 (%)	57	60	0.97
Rx (%)	2	0	0.78
Gleason score			
Gleason ≤7 (%)	84	89	0.69
Gleason >7 (%)	16	11	
Capra-S Score (median)	15.7	4	1,00
Post-operative PSA (mean, ng/mL)	0.01	0.01	1,00
bRFS (median, months)	46.3	38.4	0.11
Biochemical recurrence (%)	16	16	0.83
Follow-up (median, months)	56.5	53.6	0.56

*PSA, prostate specific antigen, MRI, magnetic resonance imaging; bRFS, biochemical relapse free-survival*.

### Outcome

Median follow-up was 49.9 months (range, 24–100.3). Among the selected 107 patients, BCR occurred in 17 patients (16%) after a median duration of 24 months (4.14–83.1 months). Median bRFS was 42.6 months (4.14–100.3 months).

Within the relapsing population and at last follow-up, 7 (41%) patients experienced a clinical and/or radiological relapse with 3 (18%) having lymph node metastasis and 4 (24%) distant metastasis. All other patients accounted for BCR alone.

### Training Set

**Figure 2:**
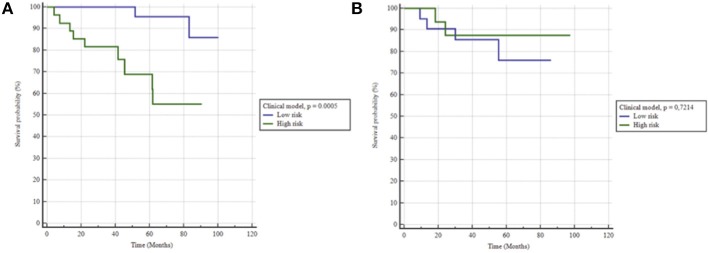
Kaplan-Meier estimates of biochemical relapse free survival using the clinical model for **(A)** training and **(B)** testing set.

Using univariate analysis, no clinical feature was significantly correlated with BCR. The most predictive model of survival without BCR was obtained with the combination of pre-operative PSA and age at surgery. The association between clinical and histopathological features and BCR are shown in the Table 3. This clinical model (age >65 y and pre-operative PSA >5.6) resulted in an AUC of 0.76 (sensitivity 82%, specificity 70%, *p* = 0.0002) and was also significantly associated with bRFS with a hazard ratio (HR) of 12.2 (*p* = 0.0005; Figure 2A). All individual ROC curves for clinical features are provided in the Supplementary Figure 3.

**Table 3:** Correlation between clinical features and biochemical recurrence.

**Clinical variable**	**Univariate analysis**			**Best cut-off**	***p-value***	**Odds-ratio**
	**AUC**	**Se**	**Sp**			
Age at surgery (y)	0.60	91	51	>65.35	0.2262	10.16
Pre-operative PSA (ng/mL)	0.60	91	39	>5.6	0.2676	6.23
Gleason score	0.65	36	90	>7	0.154	
T stage	0.62	82	34	>T2c	0.1486	
Surgical Margins	0.61	60	61	>0	0.2308	
Post-operative PSA (ng/mL)	0.64	55	71	>0.01	0.1304	
Capra-S Score	0.55	64	53	>3	0.6522	

Of note, tumor volume was not associated with BCR (AUC 0.57).

The feature set reduction technique reduced the number of radiomic features to 10 non-redundant, uncorrelated features (Supplementary Table 1), which on univariate analysis were all significantly associated with BCR (Table 4). On multivariate analysis, three of these 10 radiomic features remained strongly correlated with BCR: SZE_GLSZM_, SZLGE_GLSZM_, HGRE_GLRLM_ (feature description in Supplementary Table 1) with respective Odds-ratio of 16.6 (*p* = 0.0266), 8.8 (*p* = 0.0255), and 15.2 (*p* = 0.0111).

**Table 4:** Correlation between radiomic features and biochemical recurrence.

**Radiomic feature**	**Univariate Analysis**					**Multivariate Analysis**	
	**AUC**	**Se**	**Sp**	**Best cut-off**	***p-value***	***Odds-ratio***	***p*-value**
ADC3	0.84	91	69	≤0.528	<0.0001	16.6	0.0266
ADC6	0.79	73	81	≤0.014	0.0001	8.8	0.0255
ADC10	0.72	64	79	>93.042	0.0155	15.2	0.0111
ADC14	0.75	73	71	≤0.116	0.0005		
ADC18	0.74	82	69	≤0.067	0.0012		
ADC20	0.75	73	78	≤0.058	0.0036		
T1	0.78	91	66	≤324.593	0.0008		
T7	0.76	73	78	≤20.291	0.0009		
T10	0.80	100	59	>348.199	<0.0001		
T17	0.76	55	97	>94.004	0.0066		

*ADC, ADC MRI-scan Sequence; T, T2 MRI-scan Sequence; AUC, Area Under the Curve; Se, sensitivity; Sp, specificity*.

*Each feature description can be found in Supplementary Table 1*.

**Figure 3:**
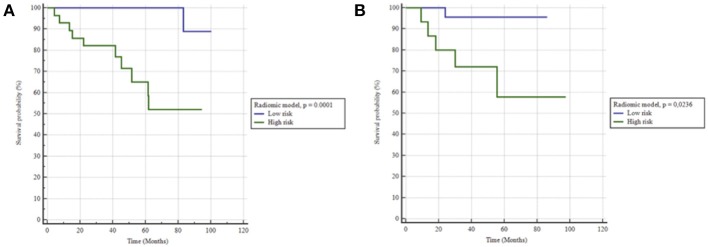
Kaplan-Meier estimates of biochemical relapse free survival using the radiomics model in **(A)** training and **(B)** testing set.

When the selected cut-off was applied (i.e., ≤0.528 for the SZE_GLSZM_ feature), no additive combination of radiomic features outperformed the ADC-based SZE_GLSZM_ feature alone with an AUC of 0.799 (sensitivity 91%, specificity 69%) and was therefore chosen for further evaluation. The model relying on this SZE_GLSZM_ feature alone resulted in strong stratification of patients for bRFS, with a HR of 17.9 (*p* = 0.0001) (Figure 3A).

All individual ROC curves for radiomic features are available in the Supplementary Figure 4.

**Figure 4:**
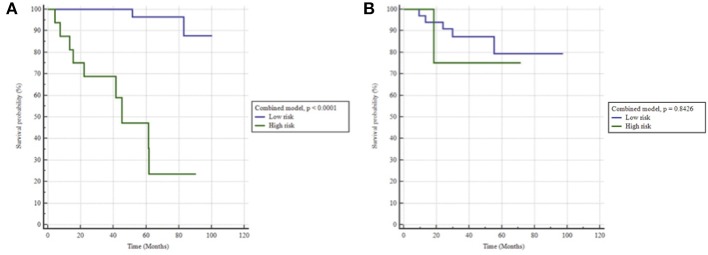
Kaplan-Meier estimates of biochemical relapse free survival using the radiomics + clinical model in **(A)** training and **(B)** testing set.

The model combining clinical (pre-operative PSA and age at surgery) and radiomic feature (SZE_GLSZM_) resulted in a high prediction of BCR with an AUC of 0.849, *p* < 0.0001 and a prediction of bRFS with a HR of 23.1, *p* < 0.0001) as shown in Figure 4.

### Testing Set

When applied to the testing set the clinical model did not hold, with an AUC of 0.57 (sensitivity 67%, specificity 47%), therefore unable to predict bRFS (*p* = 0.7) (Figure 2B). On the contrary, the radiomics-only model held well, reaching an AUC of 0.76 (sensitivity 83%, specificity 68%) and predicting rBFS with an HR of 5.1 (*p* = 0.0236) (Figure 3B). The combined radiomics-clinical model underperformed with an AUC of 0.52 only.

### Analysis According to the Type of MRI Scanner

No demographic differences were found between the two cohorts when focusing on types of MRI (Supplementary Table 2).

In the patients acquired with the Siemens 1.5T, the radiomics-only model reached an AUC of 0.76 (sensitivity 87%, specificity 66%, *p* < 0.0001), whereas in these acquired on the Philips 3T, the model had better performance with an AUC of 0.87 (sensitivity 100.00%, specificity 73%, *p* < 0.0001).

## Discussion

To our knowledge, this work is the first study investigating radiomics as a provider of potential image biomarkers to guide adjuvant treatment decision after RP.

Although none of the clinical variables were significantly predictive of BCR in the training set, combining the pre-operative PSA and age at surgery nonetheless allowed to predict BCR to an extent (AUC of 0.76). These two factors have already been reported to be prognostic for late BCR with 10 years of follow-up (18, 19). However, this clinical-only model demonstrated very low performance in the testing set (AUC 0.57). This could be partly explained by the small cohort, but also emphasizes the need for more robust predictive markers of BCR to adapt the adjuvant therapeutic strategy.

Radiomic features extracted from pre-therapeutic scans were found to have high predictive ability regarding BCR in PCa. One radiomic feature in particular, small zone emphasis (SZE_GLSZM_), remained strongly correlated to the risk of BCR, independently from the clinical variables and other radiomic features. SZE is calculated on the Gray-Level Small Zone Matrix (GLSZM). GLSZM quantifies gray level zones, defined as the number of connected voxels sharing the same gray level intensity: a homogeneous tissue will thus have large zones of same gray-level values. On the contrary, a more heterogeneous tissue will exhibit more limited zones with small distances. SZE allows focusing on areas of small zones, particularly adapted to PCa. The lower SZE's value is, the more heterogeneous the intensity distribution in the image is (15).

Recently published EAU guidelines (20) recommend to systematically discuss adjuvant radiotherapy in case of high-risk prostate cancer. If taken to an extreme, this could result in unnecessary treatment for more than 80% of patients (84% in our cohort), whereas the radiomics-based model, thanks to a predictive negative value of 96%, could allow a reduction of unnecessary treatment to 14/107 (13%) patients. This model could therefore be useful for a better selection of men eligible for aRT.

These findings are in line with several recent studies that investigated radiomics in PCa for diagnosis, prognosis and therapy. Very few studies have been published exploring the possibilities of texture analysis regarding Pca. To our knowledge, most of these studies (21, 22) implied radiomic features extracted from ADC and T2 sequences alone, these sequences being the most useful and robust sequences. Wibmer et al. evaluated MRI-derived radiomics for the detection of PCa in 146 patients (21). Four Gray level co-occurrence matrix (GLCM)-derived textural features (energy, entropy, correlation, and homogeneity) were significantly associated with the presence of PCa. Cameron et al. developed a quantitative radiomics approach for PCa detection combining all imaging sequences and aiming to improve MRI sensitivity and specificity (23). First, tumoral tissues were automatically delineated on a multiparametric MRI. The MAPS (Morphology, Asymmetry, Physiology, and Size) feature model was then used to score the candidate regions. The MAPS model outperformed all other feature sets with a sensitivity of 86%, a specificity of 88% and an accuracy of 87%.

These studies emphasize the recent development of computer-aided diagnosis solutions, waiting for larger datasets and better feature selection to be implemented on a daily basis. Exploring these new developments, a couple of studies were recently published. Based on two institutions (70 and 50 patients) and two different MRI scans, Shiradkar et al. developed a classifier based on radiomics and clinical variables with an AUC of 0.74 in the testing set (24). The main limitation of this work was that the model was trained using a cohort of patients who underwent heterogeneous treatment strategies (surgery, RT or androgen deprivation therapy), but it was then tested only on patients treated with surgery, who underwent a third type of MRI. Focusing on outcomes after RT, Gnep et al. showed the prognostic value of texture analysis after RT with androgen deprivation therapy (25). In their study, Haralick textural features derived from T2-w MRI were able to predict BCR following treatment in 74 patients after a median follow-up of 47 months, with a c-index of 0.90. However, no external validation was performed.

Interestingly, when we evaluated our radiomics model on the entire cohort, its prediction performance was higher on the subset of patients acquired with the 3T scan than the 1.5T scan (AUCs of 0.87 and 0.76, respectively). Numerous retrospective studies support the superiority of 3T over 1.5T scans when using the same type of body phased-array coil. In 2018, Ryznarova et al. showed that the best accuracy for tumor staging was obtained with a 3T MRI with DCE when compared to 3T MRI without DCE and 1.5T MRI with respective accuracy prediction scores of 90, 72, and 66% in a cohort of 103 patients (26).

Furthermore, acquisition parameters differed between the two scans especially the echo-time on T2 acquisitions and B-values on the ADC sequence, differences that we took into account when evenly dispatching patients into the training and testing cohorts.

The type of MRI scan being well-balanced in each cohort, we did not apply any *a posteriori* harmonization such as the Combat method (27), which could however be considered in future works to explore more in depth machine learning methodologies (e.g., 10-fold cross validation and alternate feature selection strategies)

Whether patients at high risk of BCR should receive adjuvant or sRT also remains a matter of debate. At present the choice between postoperative RT and early sRT should be based on a stratified risk approach in the context of a multidisciplinary meeting and according to individual patient preferences. The results of the meta-analysis of the RAVES, GETUG, and RADICALS randomized trials are expected in 2019 and will hopefully answer some of these questions. The availability of highly sensitive imaging modalities such as 68Ga-PSMA-PET will also probably change the therapeutic management of patients with a low PSA ranging between 0.2 and 0.5 ng/mL (28).

The radiomics approach applied to routinely acquired images for diagnosis has the great advantage of being cost-effective and non-invasive. Lately, recent advances in the field of genomics have led to the distribution of several genomic tests such as the Decipher Prostate Cancer test® (29). Among 256 high-risk PCa patients, the c-index of the genomic test was 0.79 (CI 95% 0.68–0.87) (30). Radiogenomics, the integration of quantitative imaging data with genomic signatures could be of interest in the field of PCa, but very few studies are available to this date.

We have to emphasize the short follow-up of our study as a potential limitation, especially in PCa. Selecting a minimal follow-up of 3 years would have resulted in a small cohort prohibiting the data analysis. However, time from RP to BCR is, on average, 3.5 years (31). Furthermore, the BCR rate is low with a rate of 16% after a median follow-up of 48.6 months. This is consistent with previous studies. For example in a cohort of 1997 men who underwent RP, and among which 25.8% had stage ≥T2b, and 40% a Gleason score ≥7, BCR occurred in 15% of patients (31).

A further analysis with a longer follow-up will definitely be needed to confirm our findings.

Moreover, the addition of other MRI sequences (such as perfusion providing with a dynamic assessment of PCa and diffusion) are currently at work in our center.

## Conclusion

A radiomics based model was trained and internally validated. It appears to be predictive of BCR and a prognostic factor of bRFS after RP in patients with high risk PCa. With a negative predictive value of 96%, this model could help identifying patients at very low risk of recurrence, allowing for a better guidance of patients eligible for aRT or those who would undergo careful watching, thus reducing the number of unnecessary treatments and associated toxicity. Exploring the correlation between these features and clinical outcome with a longer follow-up is needed and is currently under investigation in our center. In addition, we intend to validate the model in external cohorts.

## Data Availability

All datasets generated for this study are included in the manuscript/Supplementary Files.

## Ethics Statement

This study was approved by the hospital ethical committee (PREBOP 29DRC18.0108) and all patients gave their consent for the use of their clinical and imaging data.

## Author Contributions

VB is the main investigator. MH and US are the supervisors. Each other author reread and validated the main manuscript.

### Conflict of Interest Statement

The authors declare that the research was conducted in the absence of any commercial or financial relationships that could be construed as a potential conflict of interest.

## Abbreviations

aRT: Adjuvant radiotherapy
PCa: Prostate cancer
RP: Radical prostatectomy
BCR: Biochemical recurrence
R1: Positive margins
RT: Radiation therapy
sRT: Salvage radiation therapy
PSA: Prostate-specific antigen
bRFS: Biochemical relapse-free survival
MRI: Magnetic resonance imaging
IBSI: Image Biomarker Standardization Initiative
ROC: Receiver operating characteristic
PC: mean absolute Pearson's coefficient
AUC: Area under curve.
