# Supplementary material for: MRI-Derived Radiomics to Guide Post-operative Management for High-Risk Prostate Cancer
Source: Front Oncol. 2019 Aug 27;9:807. doi: 10.3389/fonc.2019.00807 (PMC6719613; doi:10.3389/fonc.2019.00807)
Supplement: Supplementary file 1 [file Data_Sheet_1.docx]

SUPPLEMENTARY MATERIAL:

**Supplementary Table 1**: Radiomic features description

| Radiomic feature | | Wavelet |
| --- | --- | --- |
| ADC3 | glszm_3D__Fszm_sze | LLL |
| ADC6 | glszm_3D__Fszm_szlge | LLL |
| ADC10 | glrlm_3D__Frlm_hgre | LLH |
| ADC14 | glrlm_3D__Frlm_lrlge | LHL |
| ADC18 | gldzm_3D__Fdzm_ldlge | HLL |
| ADC20 | gldzm_3D__Fdzm_ldlge | HHH |
| T1 | glcm_3D__Fcm_joint_var |  |
| T7 | glcm_3D__Fcm_auto_corr | LHL |
| T10 | gldzm_3D__Fdzm_hgze | LHL |
| T17 | glrlm_3D__Frlm_hgre | HLH |

Imaging sequence:

- ADC: ADC MRI-scan Sequence
- T: T2 MRI-scan Sequence

Matrix (13) :

- Glszm: Grey-Level Small Zone Matrix
- Fszm : Fuzzy Small Zone Matrix
- Glrlm : Grey Level Run-Length Matrix
- Frlm : Fuzzy Run-Length Matrix
- Gldzm :Grey Level Distance Zone Matrix
- Glcm: Grey-level Co-occurrence Matrix
- Fcm: Fuzzy Co-occurrence Matrix
- Fdzm: Fuzzy Distance Zone Matrix

Wavelet (30) :

- LLL : Low Low Low
- LLH : Low Low High
- LHL : Low High Low
- HLL : High Low Low
- HHH : High High High
- HLH : High Low High

Feature:

- Sze : Small Zone Emphasis
- Szlge : Small Zone Low Grey Level Emphasis
- Hgre : High Grey Run Emphasis
- Lrlge : Long Run Low Grey Emphasis
- Joint Var: Joint Variance
- Auto Corr: Auto Correlation
- Hgze: High Grey zone emphasis

**Supplementary Table 2**: Patients and tumors characteristics of the initial population according to the MRI type

| Patients characteristics | | Siemens | Phillips | | p |
| --- | --- | --- | --- | --- | --- |
| Number of patients | | 75 | 32 | |  |
| Mean age at diagnostic (y) | | 65.4 | 64.9 | | *0.701* |
| Mean PSA (ng/mL) | | 8.78 | 9.85 | | *0.524* |
| Surgical characteristics | | | | | |
| Post-operative tumour status (%) | pT1-pT2 | 36.0 | | 34.4 | *0.950* |
|  | pT3-pT4 | 64.0 | | 65.6 | *0.950* |
| Surgical margins (%) | R0 | 37.3 | | 50.0 | *0.314* |
|  | R1 | 62.7 | | 50.0 | *0.314* |
| Gleason score (%) | Gleason ≤ 7 | 89.3 | | 81.3 | *0.418* |
|  | Gleason > 7 | 10.7 | | 18.7 | *0. 418* |
| Median Capra-S Score | | 4 | | 4 | *1* |
| Mean Post-operative PSA (ng/mL) | | 0.012 | | 0.013 | *0.508* |
| Median bRFS (months) | | 42.6 | | 55.3 | *0.834* |
| Biochemical recurrence (%) | | 20.0 | | 6.3 | *0.137* |
| Median follow-up ( months) | | 52.1 | | 55.5 | *0.525* |

*Abbreviations: MRI= magnetic resonance imaging, T= Tesla, PSA= prostate specific antigen, bRFS=biochemical relapse-free survival*

**Supplementary Figure 1:** Examples of delineation on both T2 (left) and ADC (right) sequences. Images acquired on a Philips 3T Scan.


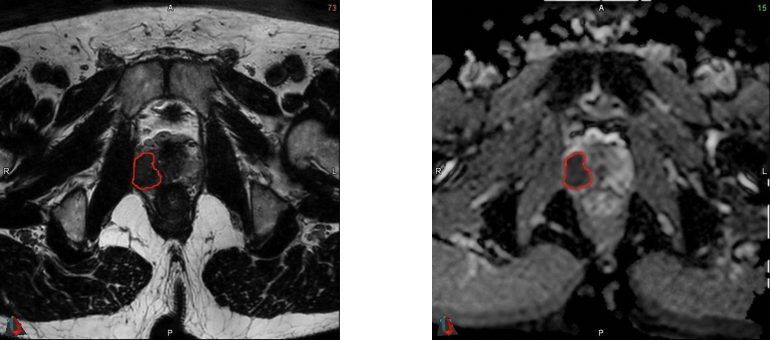


**Supplementary Figure 2:** Image processing scheme for image feature calculation. Depending on the specific imaging modality and purpose, some steps may be omitted. The IBSI defines 79 non-texture features and 94 textural features. Each of the 94 textural features were computed 32 times in each of the 9 image spaces using all possible combinations of the following different extraction parameters (a process previously named “texture optimization” (13): (i) isotropic voxel sizes of 1 mm, 2 mm, 3 mm and 4 mm; (ii) “fixed bins number” (FBN) discretization algorithms, with and without equalization; and (iii) number of grey levels of 8, 16, 32 and 64. Thus, in the case of texture features, a total of 94 × 32 × 9 = 27,072 features were created over all image spaces. Furthermore, some non-textural features were not computed due to the indefinite units of the MRI raw data (23 features for the original image space) and/or the redundancy in calculating twice some morphological features in the original and filtered image spaces (48 features for each filtered space). Thus, in the case of non-textural features, a total of 56 + 8 × 31 = 304 features were extracted over all image spaces.

**
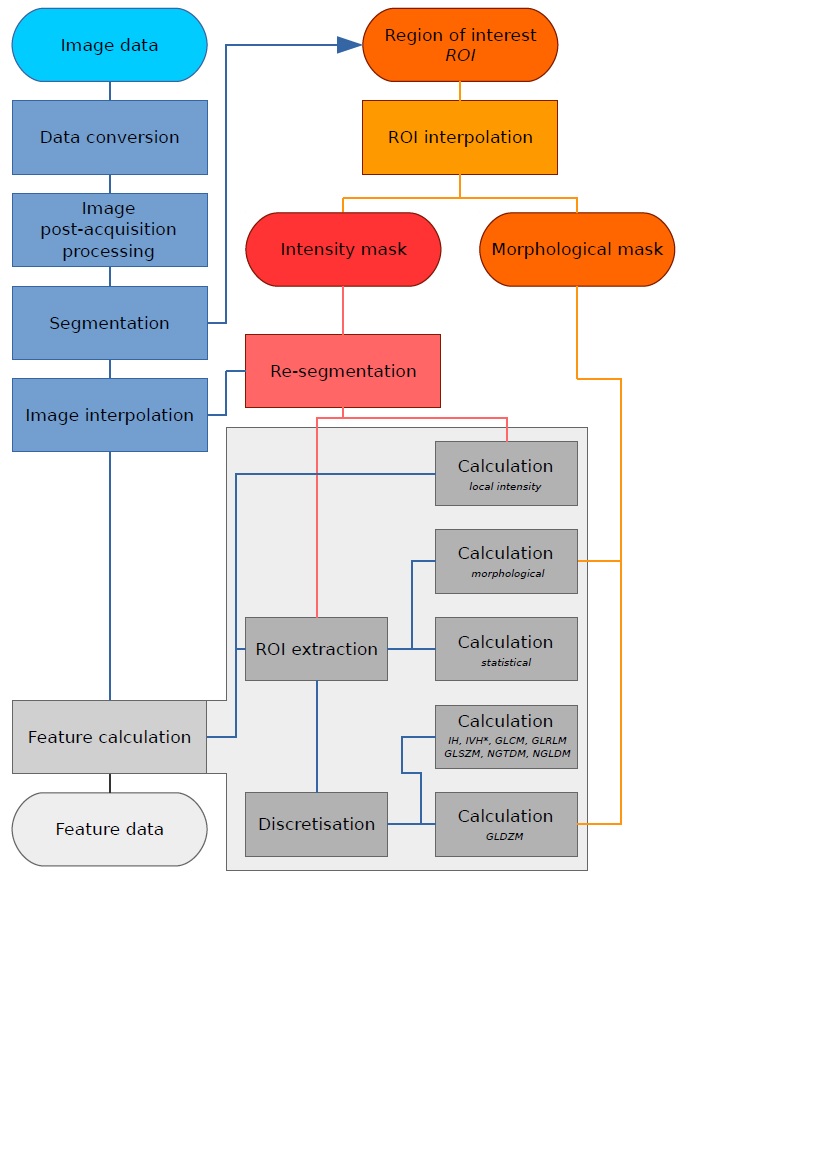
**

**Supplementary Figure 3:** Receiver operating characteristic (ROC) curves in the teaching set – clinical and histopathological Features

**A:** according to Gleason score

**B:** according to margins status

**C:** according to age at surgery

**D:** according to CAPRA-S Score

**E:** according to PSA at diagnosis

**F:** according to post-operative PSA


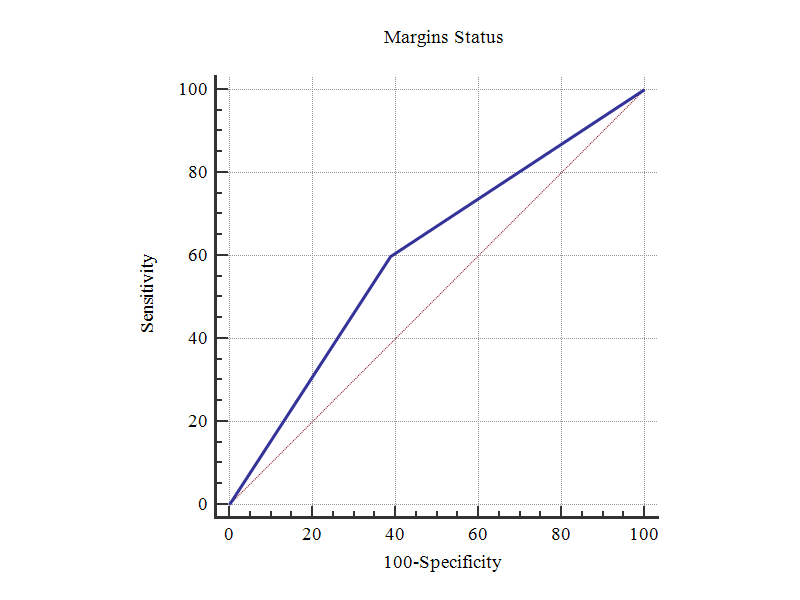


A


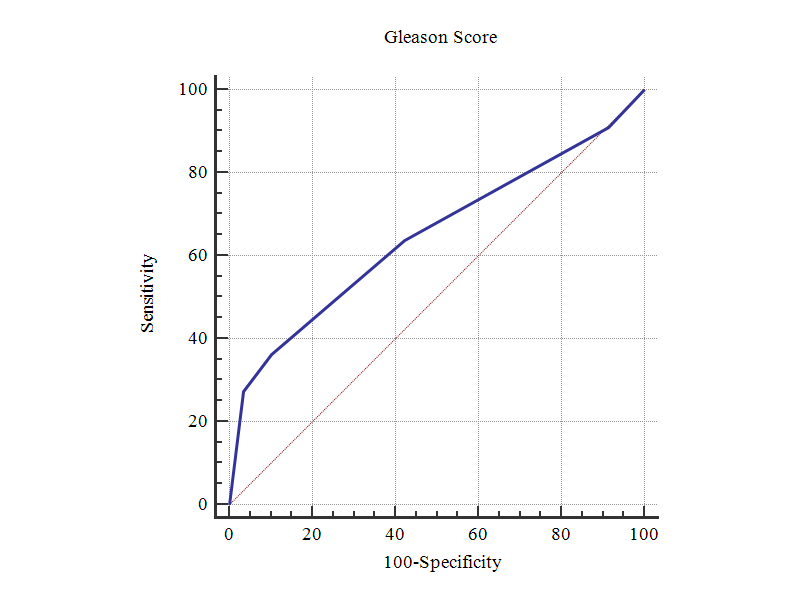

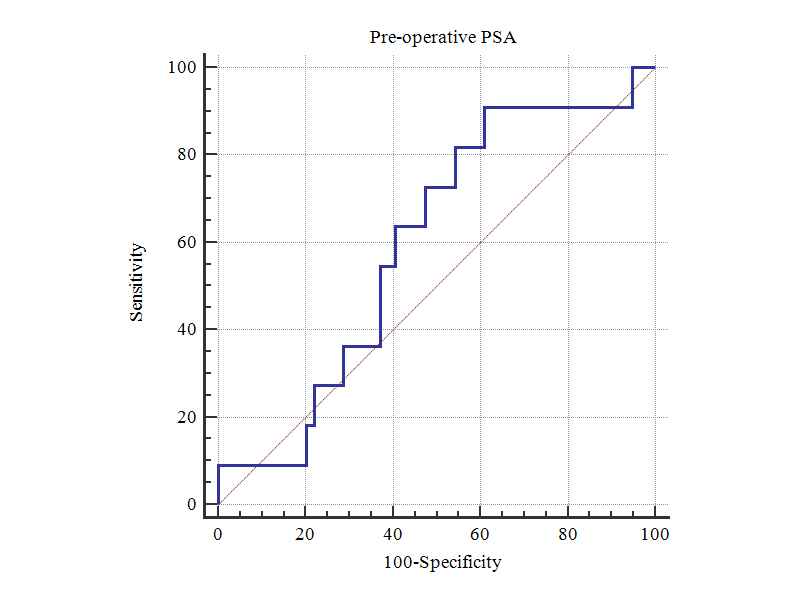

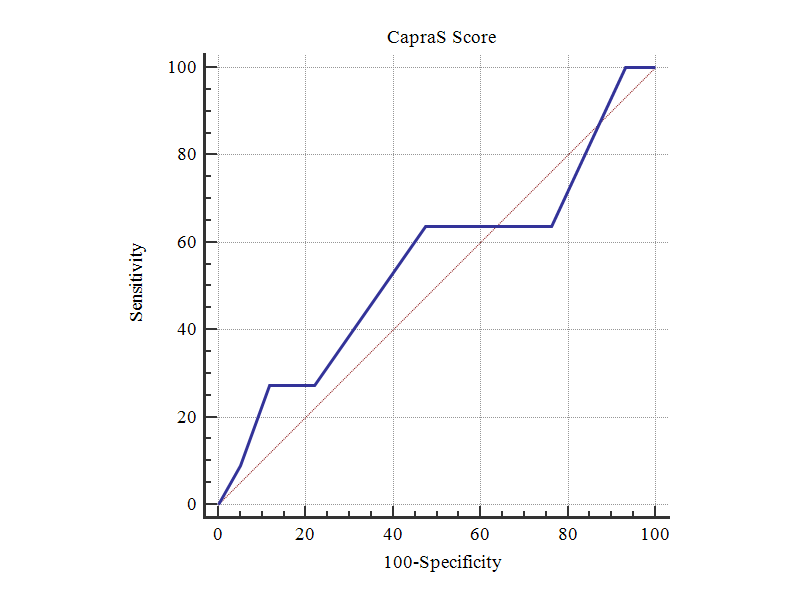

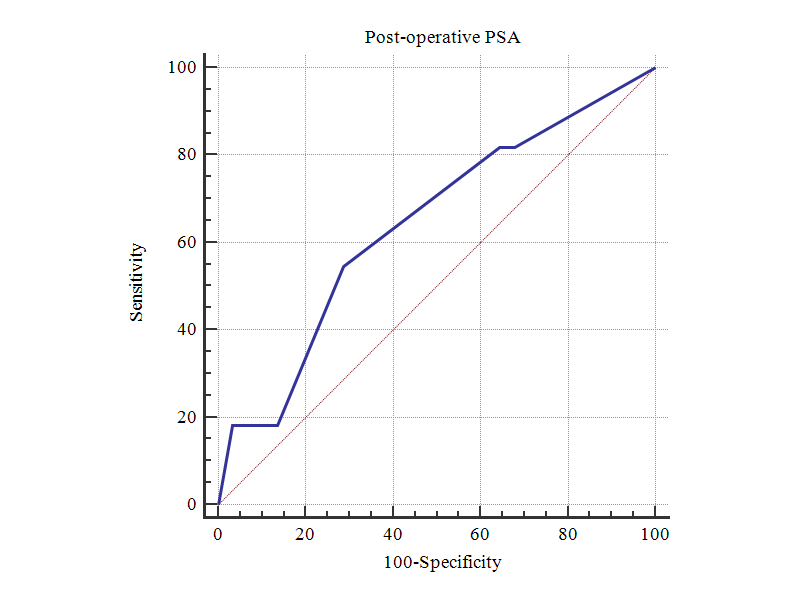

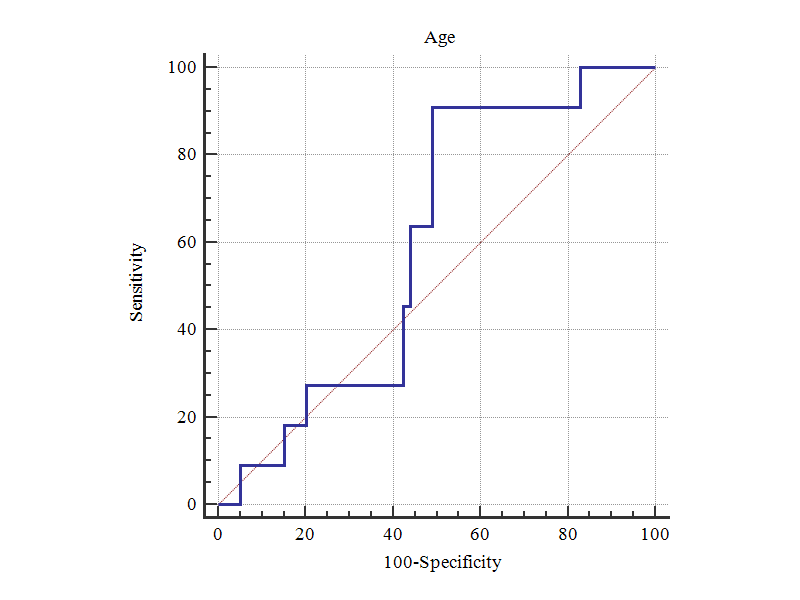


C

B

D

E

F

A

**Supplementary Figure 4:** Receiver operating characteristic (ROC) curves in the teaching set – radiomic features

**A:** according to ADC3

**B:** according to ADC6

**C:** according to ADC10

**D:** according to ADC14

**E:** according to ADC18

**F:** according to ADC20

**G:** according to T1

**H:** according to T7

**I**: according to T10

**J:** according to T17


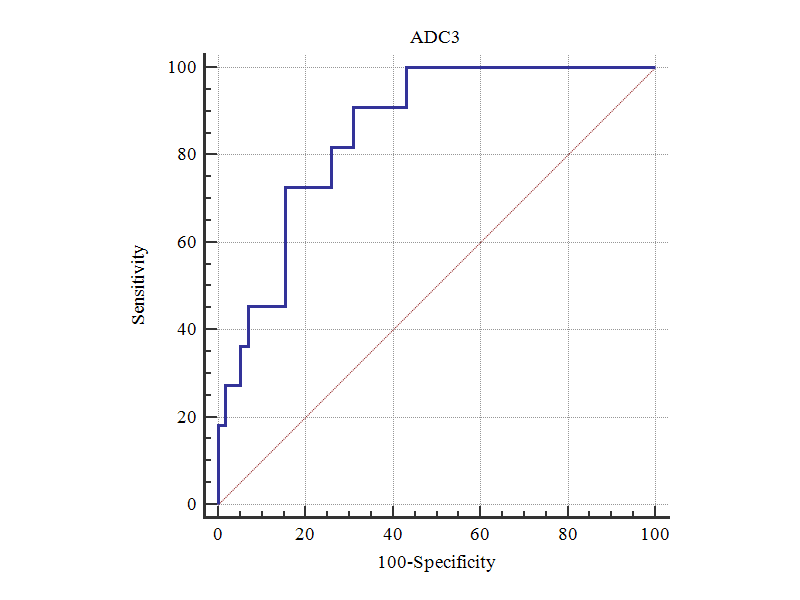

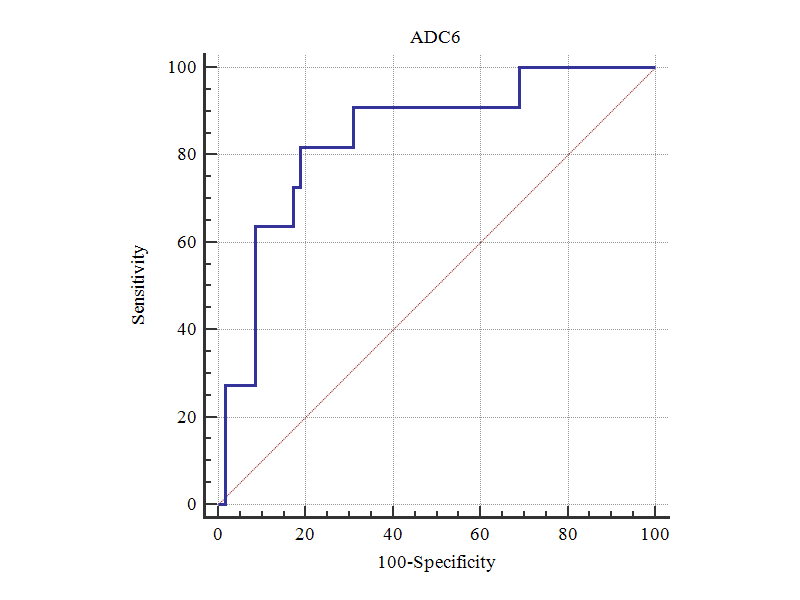

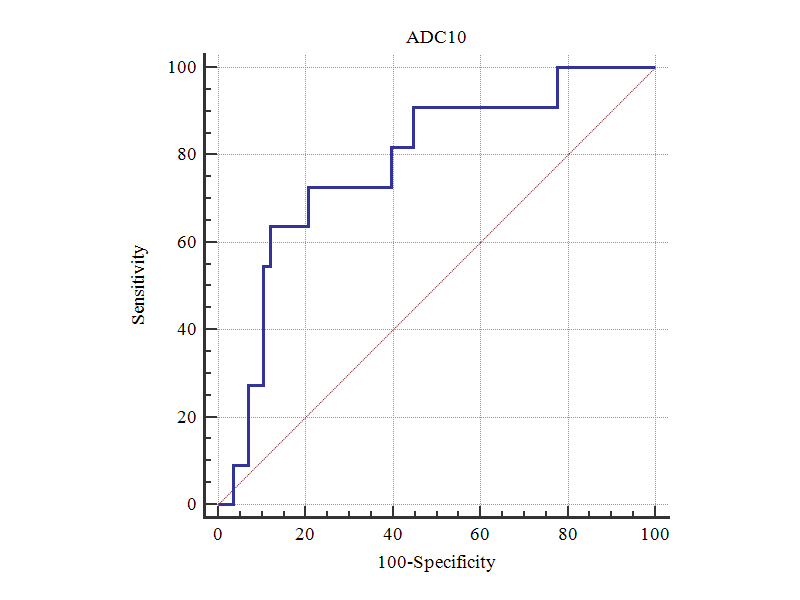

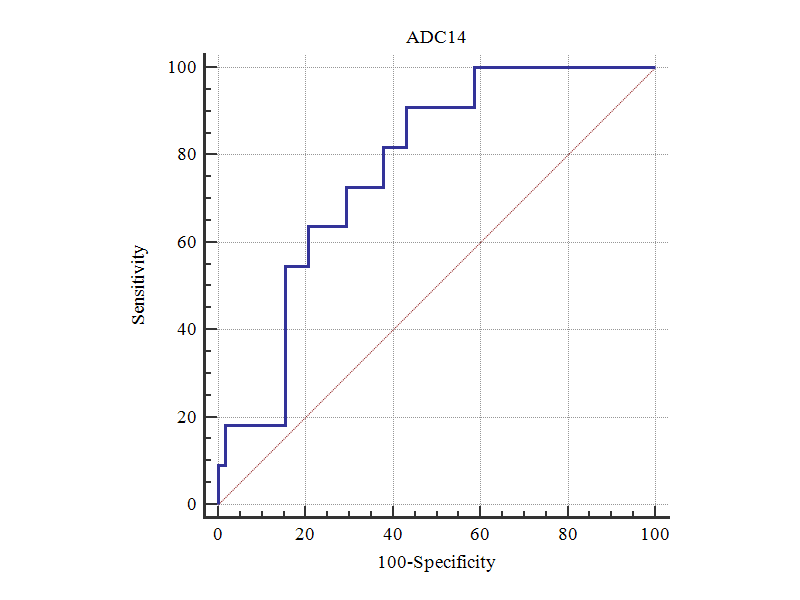

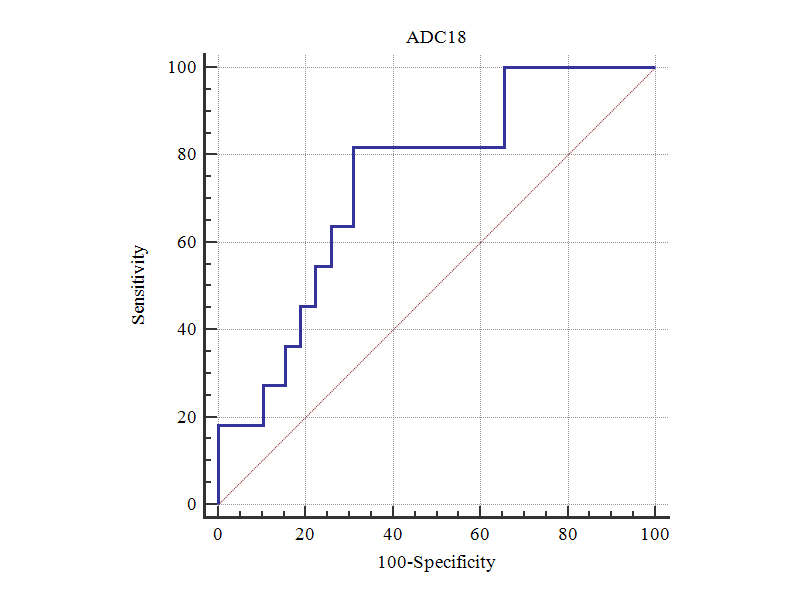

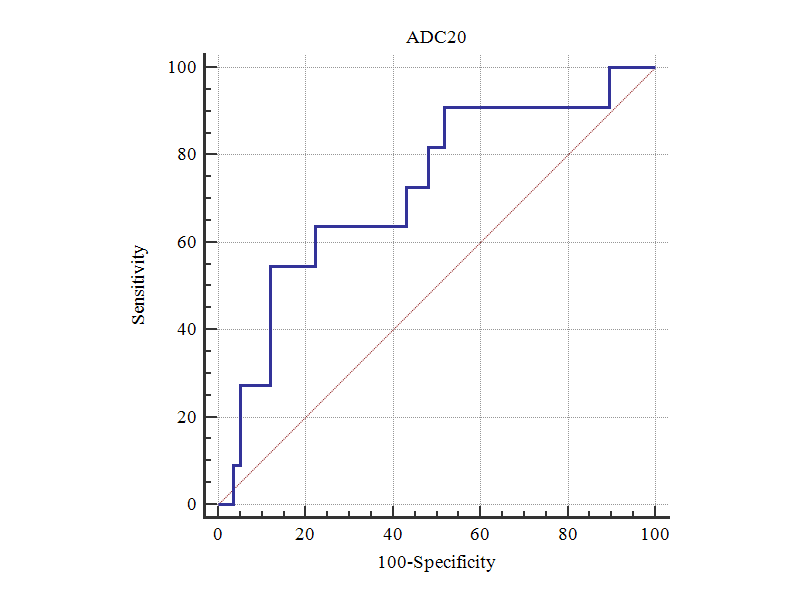

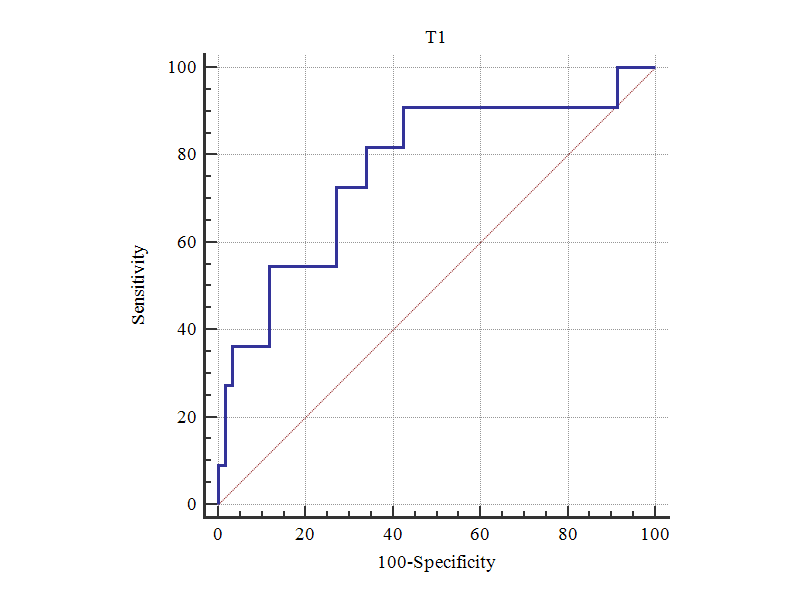

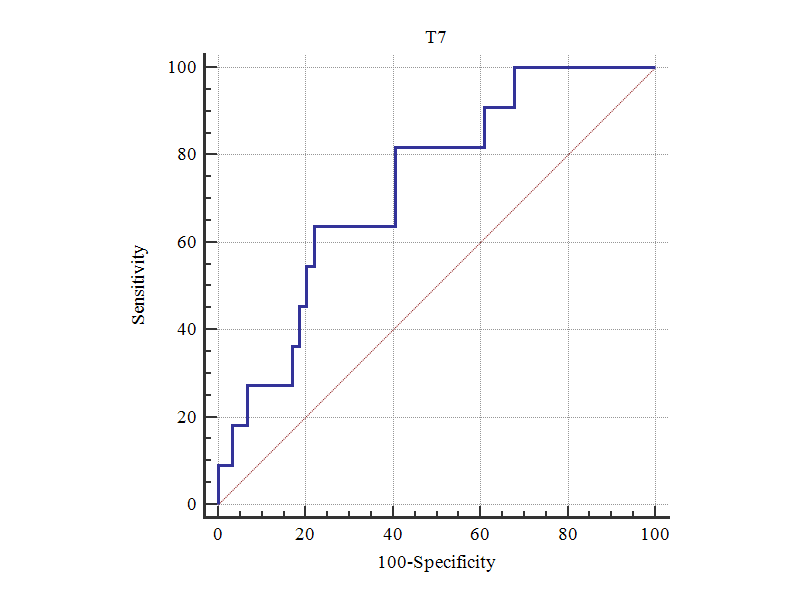

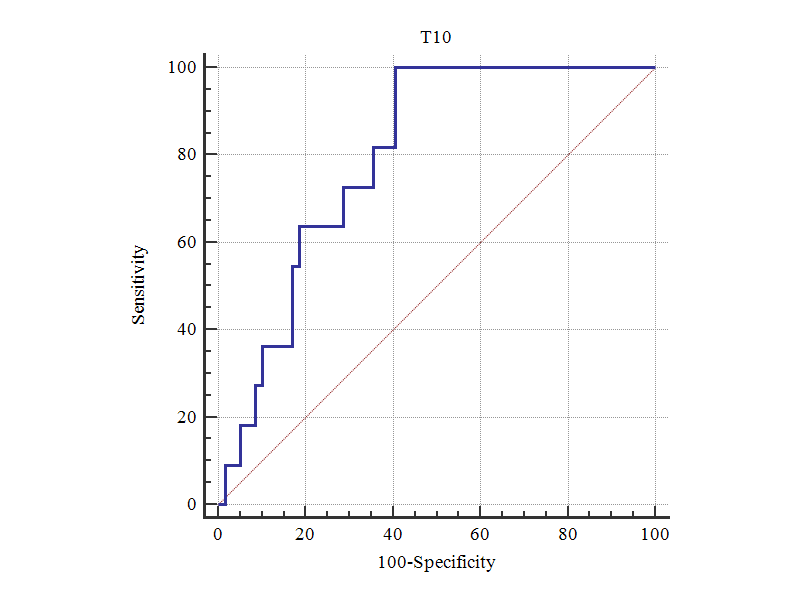

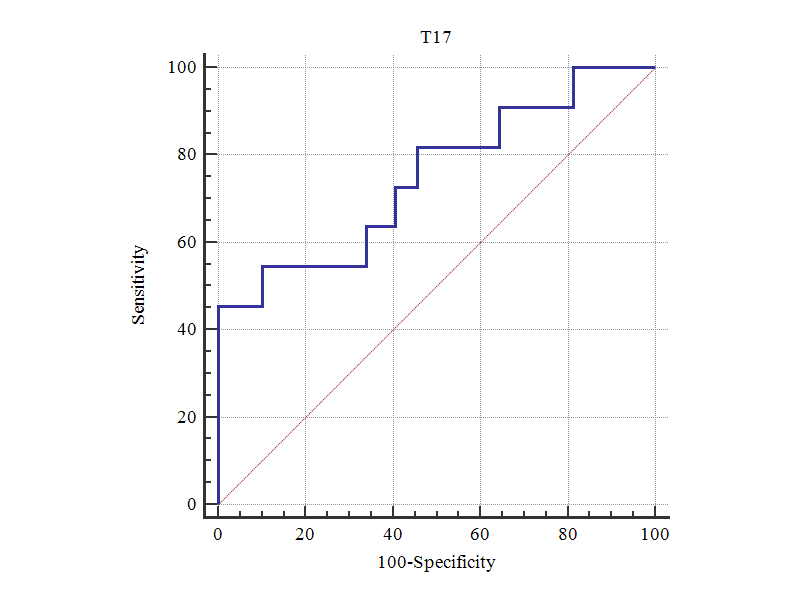


A

B

C

D

E

F

G

H

I

J
